# Antibody Competition Reveals Surface Location of HPV L2 Minor Capsid Protein Residues 17–36

**DOI:** 10.3390/v9110336

**Published:** 2017-11-10

**Authors:** Stephanie M. Bywaters, Sarah A. Brendle, Kerstin P. Tossi, Jennifer Biryukov, Craig Meyers, Neil D. Christensen

**Affiliations:** 1 Department of Microbiology and Immunology, The Pennsylvania State University College of Medicine, Hershey, PA 17033, USA; jenbiryukov@gmail.com (J.B.); cmm10@psu.edu (C.M.); 2 Jake Gittlen Laboratories for Cancer Research, The Pennsylvania State University College of Medicine, Hershey, PA 17033, USA; sab40@psu.edu (S.A.B.); ndc1@psu.edu (N.D.C.); 3 Medigene AG, 82152 Planegg-Martinsried, Germany; k.pino-tossi@medigene.com

**Keywords:** HPV, capsid, structure, L2, minor capsid protein, mAb, competition

## Abstract

The currently available nonavalent human papillomavirus (HPV) vaccine exploits the highly antigenic L1 major capsid protein to promote high-titer neutralizing antibodies, but is limited to the HPV types included in the vaccine since the responses are highly type-specific. The limited cross-protection offered by the L1 virus-like particle (VLP) vaccine warrants further investigation into cross-protective L2 epitopes. The L2 proteins are yet to be fully characterized as to their precise placement in the virion. Adding to the difficulties in localizing L2, studies have suggested that L2 epitopes are not well exposed on the surface of the mature capsid prior to cellular engagement. Using a series of competition assays between previously mapped anti-L1 monoclonal antibodies (mAbs) (H16.V5, H16.U4 and H16.7E) and novel anti-L2 mAbs, we probed the capsid surface for the location of an L2 epitope (aa17–36). The previously characterized L1 epitopes together with our competition data is consistent with a proposed L2 epitope within the canyons of pentavalent capsomers.

## 1. Introduction

Human papillomaviruses (HPVs) are epitheliotropic viruses commonly thought to begin their infectious pathway through micro-abrasions in the skin or mucosae. Over 170 different HPV types have been fully sequenced [1] and subsets of mucosal types are further defined as “high-risk” or “low-risk” types. The low risk types are associated with benign skin warts of the hands, feet and genitals. The high-risk types are known to induce cervical cancer and are also implicated in cancers of the mouth, oropharynx, vagina, anus and penis [2]. The two HPV types responsible for the greatest number of cervical cancer cases are HPV16 and HPV18 and thus the research field has largely focused on these two high-risk types [2]. The 8 kb double stranded DNA genome is encapsidated by a non-enveloped T = 7 icosahedral capsid approximately 55 nm in diameter. The capsid itself is composed of only two capsid proteins, the major capsid protein L1 and the minor capsid protein L2.

Although there is currently a prophylactic vaccine available which offers protection against HPV types 6, 11, 16, 18 and a nonavalent vaccine which includes, in addition, types 31, 33, 45, 52, and 58, cervical cancer continues to be a global health burden [3]. These vaccines consist of virus-like particles (VLPs) formed using only the major capsid protein, L1. The dominant immunological response against HPV typically elicits neutralizing antibodies against the type-unique hypervariable loops of the major capsid protein [4,5,6,7,8]. Numerous studies have examined the L2-specific antibody response to L2 peptide or intact particles. Such studies have demonstrated that the anti-L2 antibodies are protective and offer cross-neutralizing potential [9,10,11,12,13,14,15,16,17,18,19,20,21]. Regional conservation of L2 makes the minor capsid protein an excellent target for vaccine development and current efforts continue to work towards such a prophylactic vaccine as reviewed by Jiang et al. [22]. 

While virus-like particles (VLPs) can be synthesized using only L1, particles encapsidating genomes are more efficiently made in the presence of L2 [23,24,25,26]. L2 also plays a role in viral infection and entry. The mechanism of viral entry continues to be a strong area of investigation and there are currently two competing models [27,28]. Both models agree that interaction between HSPGs and L1 triggers conformational changes on the viral capsid surface. However, raft-derived HPV16 has been shown to enter cells via a glycosaminoglycan-independent mechanism [29]. These structural changes expose previously buried L2 epitopes sensitive to the proprotein convertase, furin, which cleaves L2 at the amino terminus. Cleavage may facilitate interactions with additional cellular receptors followed by viral entry via endocytosis. The role of the minor capsid protein is not restricted to entry; the protein also serves to deliver the viral genome to the nucleus. Several studies have implicated L2 in the genome’s escape from the endosome, trafficking to the nucleus by way of interactions with the cytoskeleton, and also in nuclear entry during mitotic events (reviewed in Wang et al. [30]).

The exact ratio of the L1 and L2 proteins is also debated. Early investigations used SDS-PAGE and immunoblots to estimate the ratios, but results ranged from 12 to 72 L2 monomers per capsid [26,31,32,33]. As technology advanced, several investigators examined the capsid structures of HPV, bovine papillomavirus (BPV) and cottontail rabbit papillomavirus (CRPV) via cryo-electron microscopy (cryo-EM) [34,35,36,37,38,39]. Early cryo-EM studies failed to observe any density attributable to the minor capsid protein [35]. A more recent study produced pseudovirus particles in a system where L2 was over-expressed and particles were examined by cryo-EM [38]. L2 localization was assessed via a difference map where the density from the L1-only reconstruction was subtracted from the L1/L2 reconstruction to reveal the density only attributable to L2. A total of 72 buttons of density at the center of each pentamer was observed, allowing the investigators to conclude that a maximum of 72 L2 monomers can be incorporated into the capsid [38]. Currently, the amount of incorporated L2 remains controversial and recent attempts (including our own) at reconstructing particles tagged by anti-L2 monoclonal antibodies (mAbs) have failed likely due to antibody affinity, low L2 occupancy, limited L2 exposure, heterogeneous incorporation of L2 into different particles or an asymmetric distribution which dissolves the L2 density following icosahedral averaging [30]. 

The location of L2 as a target for neutralizing antibodies becomes important in the wake of new vaccine strategies targeting L2 [40]. Prior to implementation of an L2 vaccine, we need to be sure that the diagnostic tests in place to assess patient responsiveness to vaccines will not be compromised by mAb interference. Antibodies targeting the N-terminus of L2 have previously been described such as RG-1 and K4 [11,41], but it cannot be assumed that all mAbs targeting the same minimal epitopes have the same behavior. In this study, we report on the characterization of two novel anti-L2 mAbs produced by immunizing mice with recombinant Adeno-associated virus like-particles concurrently displaying the HPV16 and HPV31 L2 aa17–36 epitopes [42]. Although previous attempts by imaging have led to inconclusive results concerning the amount and location of L2 incorporation, our goal in this study was to use combinations of anti-L1 and novel anti-L2 mAbs as unbiased probes to further characterize L2 localization. Our findings indicate that the epitopes of anti-L1 mAb H16.U4 (U4) and the anti-L2 epitopes of mAbs H16.L2.2E (2E), H16.L2.1A (1A), and RG-1 are located in adjacent areas within the canyons of pentavalent capsomers.

## 2. Materials and Methods

### 2.1. Generation of L2 mAbs

Recombinant Adeno-Associated Virus engineered to display amino acids near the N-terminus of HPV16 L2 (AA 17–36) on the surface of its capsid [42] (kindly supplied by Medigene) was used to immunize BALB/c mice as previously described [16,43]. The resultant hybridomas were adapted to animal component-free medium (Sigma-Aldrich, St. Louis, MO, USA) and two HPV16 positive mAbs (16.L2.1A and 16.L2.2E) were selected. Cell supernatants were collected and either diluted or purified via protein A (Pierce) for studies. Isotype switch variants were generated for H16.V5 (IgG2b switched to IgA) and 2E (IgG1 switched to IgG2b). The hybridomas were prepared by cloning and selection of isotype-specific switch variant hybridoma cells by previously published methods [44]. 

### 2.2. PsV/QV Particle Production and Purification

Pseudovirus (PsV) and Quasivirus (QV) particles were produced by transfecting 293TT cells with HPV16 sheLL plasmids (a gift from the Schiller Lab, NCI, Bethesda, MD, USA) together with either the plasmid for secreted alkaline phosphatase (pSEAP) or cottontail rabbit papillomavirus (CRPV) genome respectively as previously described [23,24,45,46,47]. Two days post-transfection, cells were collected and lysed using the detergent Brij58 (Sigma-Aldrich, St. Louis, MO, USA). Particles were matured by incubating cell lysates at 37 °C for 24 h and subsequently treating with Benzonase (Sigma-Aldrich, St. Louis, MO, USA) and Plasmid Safe (Epicentre, Madison, WI, USA). Purification of the resulting particles was performed by ultracentrifugation of the lysates at 234,000× *g* for 3.5 h on an Optiprep step gradient (Sigma-Aldrich, St. Louis, MO, USA). Particles were harvested by puncturing the bottom of the centrifuge tube and collecting fractions of ~250 µL.

### 2.3. Production and Isolation of Native HPV in Organotypic Raft Culture

Production of native HPV virions was done by growing immortalized HPV-containing keratinocytes in organotypic raft cultures as previously described [48,49]. Briefly, cell lines were seeded onto collagen matrices containing rat-tail type 1 collagen and J2 3T3 feeder cells. Following attachment and growth to confluence, the matrices were lifted onto a metal support grid and fed with E-medium supplemented with 10 μM 1,2-dioctanoyl-*sn*-glycerol (C8:0; Sigma Chemical Company, St. Louis, MO, USA) via diffusion from below. Raft cultures were allowed to stratify and differentiate for 20 days, as mature virus has been shown to be present at this time. Then, 20-day organotypic raft tissue was harvested and Dounce homogenized in phosphate buffer (0.05 M Na-phosphate, pH 8.0) as previously described. Post homogenization, removal of un-encapsidated genomes was accomplished by adding MgCl_2_ to a final concentration of 2 mM with 375 U of benzonase and incubating at 37 °C for 1 h. Samples were adjusted to 1 M NaCl and centrifuged for 10 min at 4 °C and 10,000× *g* to remove cellular debris. Virus preparations were stored at −80 °C.

### 2.4. Titration of Native HPV by qPCR

Viral titers were measured as previously described [29,50,51,52]. Briefly, viral genomes were released by re-suspension in 200 μL HIRT DNA extraction buffer (400 mM NaCl/10 mM Tris-HCl [pH 7.4]/10 mM EDTA, [pH 8.0]), 2 μL 20 mg/mL proteinase K, and 10 μL 10% SDS for 2 h at 37 °C. Following extraction, DNA was purified via phenol-chloroform extraction and ethanol precipitated overnight at −20 °C. Viral genomes were quantified using the Thermo Maxima SYBR Green qPCR kit (ThermoFisher Scientific, Waltham, MA, USA). Amplification of the HPV16 or HPV18 E2 open reading frame was performed with previously utilized HPV type specific primers. Amplification of the E2 open reading frame of serially diluted pBSHPV16 or pBSHPV18 DNA, ranging from 10^7^ to 10^4^ genome copies/μL served to generate a standard curve. A Bio-Rad (Hercules, CA, USA) iQ5 Multicolor Real-Time qPCR machine and software were utilized for PCR amplification and subsequent data analysis.

### 2.5. Sequencing of L2 mAb Variable Regions

The variable regions of H16.L2.2E and H16.L2.1A heavy and light chains were sequenced as previously published [53,54,55]. Briefly, RNA was extracted from the hybridomas using Trizol Reagent (Life Technologies, Carlsbad, CA, USA). The RevertAid first-strand cDNA synthesis kit (ThermoFisher Scientific) with the provided random primers was used to synthesize complementary DNAs (cDNAs). Resultant cDNAs were subsequently used as templates for PCR and mouse isotype specific primers previously described by Wang et al. [56] were used for amplification. PCR products were purified and sequenced with the same primers. 

### 2.6. Immunofluorescence Assay

293TT cells were seeded onto poly-l-lysine treated coverslips in a 12-well cell culture plate and grown in Dulbecco’s Modified Eagle Media (DMEM). Following incubation at 37 °C for 24 h, the cells were transfected with HPV sheLL vectors (gifted by the Schiller lab, NCI, Bethesda, MD, USA or purchased from Addgene, Cambridge, MA, USA) using Lipofectamine 2000 (ThermoFisher Scientific) and incubated at 37 °C for an additional 48 h. Coverslips were rinsed with phosphate buffered saline (PBS) and fixed using cold acetone/methanol at a 1:1 ratio. Following fixation, coverslips were blocked with 2% bovine serum albumin (BSA) in PBS with 0.05% Tween 20 (PBS/T) and a 1:100 dilution of the anti-L2 or anti-L1 supernatant was added to the coverslips. Coverslips were washed and Hoescht and fluorophore labeled secondary antibody goat anti-mouse 488 IgG (ThermoFisher Scientific) were added. After additional washes in PBS/T, the coverslips were mounted to slides and visualized using a Nikon Eclipse E600 microscope (Nikon, Minato, Tokyo, Japan). Images were prepared and merged using Adobe Photoshop (Adobe Systems Inc., San Jose, CA, USA). 

### 2.7. Direct Binding ELISAs

PsV (500 ng) was bound to the wells of microtiter plates in PBS. The wells were washed with PBS/T to remove unbound particles and subsequently blocked with 5% dry milk protein in PBS/T. Next, the primary mAb (anti-L1 or anti-L2) was serially diluted and added to the wells in blocking buffer. A second primary mAb was added to the wells at a constant concentration (66.6 nM) followed by an anti-isotype specific alkaline phosphatase (AP) conjugated secondary antibody (Southern Biotech, Birmingham, AL, USA) at a 1:3000 dilution. The wells were developed in 4-Nitrophenyl phosphate disodium salt hexahydrate (pNPP) (Sigma-Aldrich, St. Louis, MO, USA) and the optical density was determined by absorbance spectrometry at optical density (OD) 405/450.

### 2.8. Peptide Binding ELISAs

L2 peptides aa17–36 of HPV types 16,18,31,35,39,45,58 and 59 (purchased from China Peptides, Shanghai, China) were bound to the wells of a 96-well microtiter plate at 1 µg/well in 50 mM sodium carbonate pH9.6 protein binding buffer. The wells were subsequently washed and then blocked with blocking buffer. Purified anti-L2 mAbs (0.5 µg) were added to the wells and binding was detected via an AP-conjugated anti-mouse IgG (H + L) secondary antibody (Southern Biotech, Birmingham, AL, USA). The OD at 405 nm was determined following addition of pNPP. 

### 2.9. Capture ELISAs

Purified anti-L1 or anti-L2 antibody (0.25 µg) was added to the microtiter plate in 50 µL of 50 mM sodium carbonate pH9.6 protein binding buffer and allowed to bind overnight at 4 °C. Following binding, the wells were washed and blocked as described previously. During this blocking step, 500 ng PsV particles per well were incubated in blocking buffer with serial dilutions of anti-L1 or anti-L2 mAb. Particles were subsequently added to the blocked wells and incubated for one hour. Unbound PsV-mAb complexes were removed by washing and then anti-L1 mAb H16.V5 (V5) isotype IgA was added for the detection of bound PsV. Following additional washes, isotype-specific AP conjugated secondary antibody was added and the signal was developed and determined as described above. 

### 2.10. QV Post-Attachment Neutralization

RA2LT cells were seeded at 1.0 × 10^5^ per well in DMEM and incubated in 12-well plates at 37 °C for 24 h [57]. Post-attachment neutralization assays required incubations of QV16 with RA2LT cells at 4 °C to allow viral binding but prevent viral entry. Unbound virus was removed by washing with DMEM. Anti-L2 mAbs were added at 4 °C and then the cells were transferred to 37 °C. Neutralization assays were harvested 72 h post-infection with TRIzol reagent (Life Technologies), RNA was extracted, and infectivity was assessed by measuring viral E1^E4 transcripts with quantitative real-time (qRT)-PCR as previously described [17,58,59] with a few modifications as follows: The Brilliant II Mastermix kit (Agilent Technologies, Santa Clara, CA, USA) was used for the qRT-PCR reactions. The following cycling conditions were applied: 50 °C for 30 min (reverse transcription), 95 °C for 10 min, and 40 cycles of 94 °C for 15 s and 60 °C for 1 min. At the end of each amplification cycle, three fluorescence readings were detected. The level of TATA binding protein was measured as an internal control. Analysis of the amplification efficiencies was performed using REST software [60].

### 2.11. Native HPV Neutralization Assay 

HaCaT cells were seeded 5.0 × 10^4^ per well in DMEM supplemented with 10% (*v*/*v*) FBS, 0.025 mg/mL gentamycin, and 0.11 mg/mL sodium pyruvate and incubated in 24-well plates at 37 °C for 48 h. Post-attachment neutralization assays required incubation of HPV16 or HPV18 with HaCaT cells at 4 °C. Unbound virus was removed by washing with PBS. Anti-L2 mAbs were added at 4 °C and then cells were transferred to 37 °C. Infections were harvested 48 h post-infection and mRNA was extracted using the QIAgen RNeasy kit (QIAGEN, Hilden, North Rhine-Westphalia, Germany). Infectivity was analyzed as previously described using a qRT-PCR based assay detecting levels of the E1^E4 splice transcript [29,50,51,52]. The level of TATA binding protein was measured as an internal control. Amplifications were performed in duplicate for each sample in 25 μL total reaction volume in 96-well qPCR plates. The Quantitect probe RT-PCR kit (QIAGEN) and the CFX-96 instrument (Bio-Rad, Hercules, CA, USA) were used to amplify the target sequences. The cycling conditions were applied: 50 °C for 30 min, 95 °C for 15 min, followed by 42 cycles of 94 °C for 15 s and 54.5 °C for 1 min. Relative levels of viral transcripts were determined using the Bio-Rad software. 

### 2.12. Quantitation of L2 Protein

The Quantitative Dot Blot (QDB) was conducted as described by Tian et al. [61]. Briefly, PsV16 was serially diluted in a denaturing loading buffer and samples were placed in a boiling water bath for 10 min. Samples were subsequently applied directly to the nitrocellulose membrane of QDB plates and allowed to dry. Membranes were briefly washed with transfer buffer and then blocked with 5% non-fat milk in PBS/T for 1 h. The wells of a flat bottom 96-well microtiter plate were filled with 100 µL primary antibody (anti-L1 or anti-L2) diluted 1:100 or 1:50 in blocking buffer and the QDB plate was fitted inside the microtiter plate. The QDB plate was incubated with primary antibody overnight at 4 °C. Membranes were washed several times with wash buffer and incubated for 1 h with 100 µL Horseradish Peroxidase conjugated Rabbit Anti-Mouse IgG (H + L) secondary mAb (ThermoFisher). Membranes were washed several more times with wash buffer and then incubated for 2 minu in 100 µL SuperSignal West Pico Chemiluminescent Substrate (ThermoFisher) prepared as according to the manufacturer. Excess substrate was shaken from the membrane and the QDB plate was placed inside a white microtiter plate. Luminescent signal was read with a microplate reader. 

## 3. Results

### 3.1. Sequencing Confirms L2 mAbs Are Separate Clones

**Figure 1:**
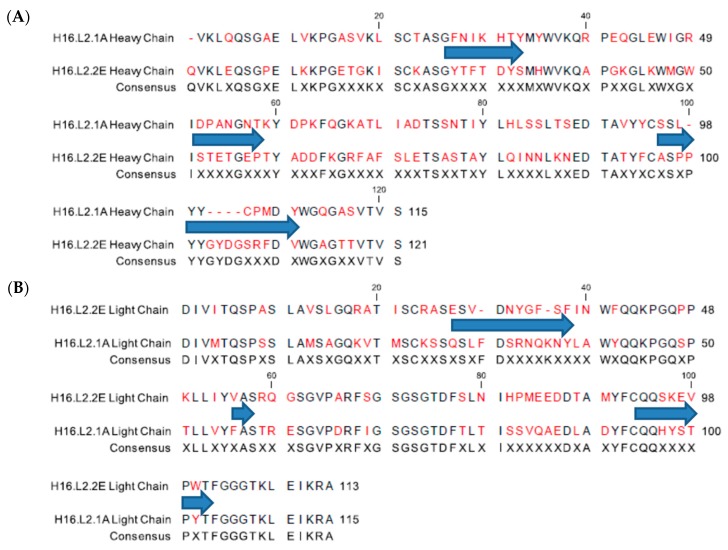
Sequence alignment of the H16.L2.2E and H16.L2.1A (**A**) heavy chain and (**B**) light chain variable regions. Sequences were aligned using CLC Main Workbench (QIAGEN, Redwood City, CA, USA) and complementarity determining regions (CDRs) were determined using IMGT/V-QUEST). The complementarity determining regions are demarcated by blue arrows. A consensus sequence is displayed below the alignment and residues highlighted in red indicate amino acid differences between the two sequences.

To ensure that the two L2 mAbs (H16.L2.1A (1A) and H16.L2.2E (2E)) generated from the chimeric Adeno-Associated Virus [42] were not derived from the same B-cell clone in the immunized mice, the variable region of each antibody was sequenced. The complementarity determining regions (CDRs) of each mAb were assessed by the international ImMunoGeneTics information system V-QUEry and STandardization (IMGT/V-QUEST) [62,63] and sequences were aligned (Figure 1). Sequencing confirmed that the two mAbs have unique sequences. As expected, the majority of variability was detected in the CDRs. The greatest difference was observed in CDR3 of the heavy chains where the sequence of 1A is considerably shorter due to deletions. There were also considerable differences present in the framework regions of both heavy and light chains. 

### 3.2. L2 mAbs Demonstrate Cross-Reactive Binding 

**Figure 2:**
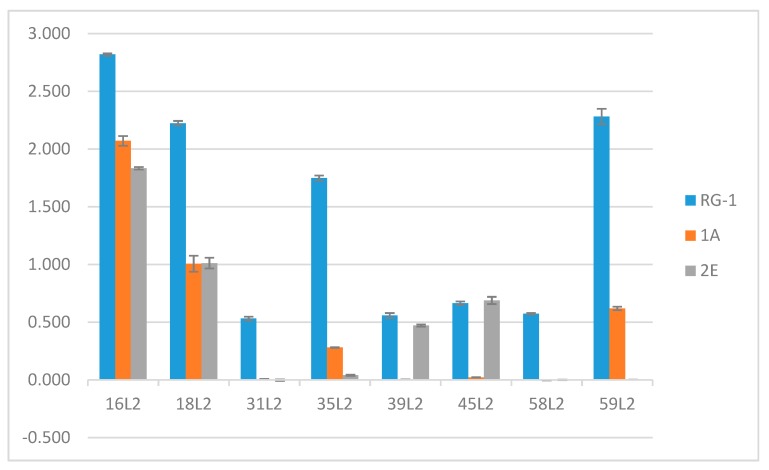
Anti-L2 mAbs differentially bind L2 peptides of human papillomavirus (HPV) type 16, 18, 31, 35, 39, 45, 58, 59 by enzyme-linked immunosorbent assay (ELISA). All L2 peptides were tested against RG-1, H16.L2.1A, and H16.L2.2E. Purified mAbs were tested in duplicate wells against peptide pre-absorbed to the wells of the microtiter plate in a peptide binding buffer. Antibody binding was determined by an anti-mouse secondary antibody conjugated to alkaline phosphatase. The mean optical density ± standard error at 405 nm is displayed in the graph.

Several regional sequences of the L2 minor capsid protein are thought to be well conserved among HPV types possibly due to the important roles these regions play in virus entry. We assessed the ability of the two L2 specific mAbs to bind the L2 peptides corresponding to aa17–36 of various HPV types (16, 18, 31, 35, 39, 45, 58, and 59). Enzyme-linked immunosorbent assays (ELISAs) demonstrated that 1A and 2E do not discriminate between the L2 peptides of HPV16 or HPV18 (Figure 2). Although the immunogen used for the production of these antibodies incorporated peptides from both HPV16 and 31 L2 [42], both antibodies failed to recognize the L2 peptides of HPV31 as well as HPV58. Interestingly, differential levels of detection were observed for HPV35, 39, 45, and 59. 2E bound weakly to HPV39 and HPV45 L2 but failed to recognize HPV35 and HPV59 L2 peptides. In sharp contrast to 2E, the 1A mAb bound HPV35 and HPV59 L2 peptides but failed to detect HPV39 and HPV45 peptides.

**Figure 3:**
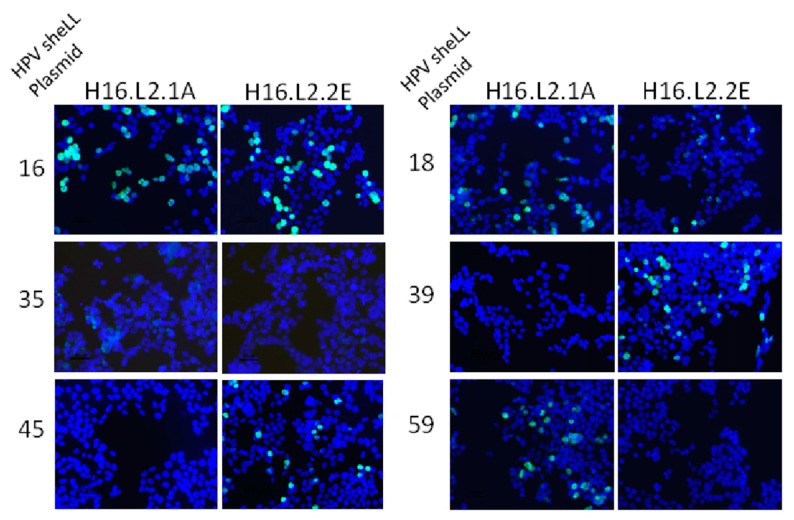
Immunofluorescence detection of anti-L2 mAb binding to L2 protein expressed in 293TT cells. Cells were transfected with sheLL plasmids for HPV16, 18, 35, 39, 45, 59 and fixed/permeabilized 48 h later. Anti-L2 mAbs were added to the cells and recognition of the L2 protein was detected with a goat anti-mouse immunoglobulin G (IgG) secondary antibody conjugated to Alexafluor 488.

Binding was also assessed in an immunofluorescence assay in which 293TT cells were transfected with a smaller panel of HPV L1/L2 plasmids (16, 18, 35, 39, 45, 59) (Figure 3). The L2 protein was detected with either 1A or 2E mAbs. The observed fluorescence confirmed the binding activity detected by peptide ELISA for most mAb/HPV genome combinations. Type specific anti-L1 mAbs confirmed L1 protein expression and transfected cells incubated only with 488 conjugated anti-mouse mAb served as a negative control (Figure S1). The specificity of the mAbs for the L2 protein was confirmed by a lack of detection in cells transfected with the HPV16 L1 expression plasmid and the absence of mAb signal when L1 VLPs were scanned by ELISA (Figures S2 and S3). We were interested to see if any residues in particular were responsible for discrimination between the two anti-L2 mAbs observed in both binding assays. The 17–36 L2 peptides from each HPV type tested were aligned but there were no obvious residues which contributed to mAb binding or a lack of mAb binding. Binding data and the L2 amino acid sequences for each HPV type are summarized in Table 1. 

### 3.3. L2 mAbs Are Capable of Neutralizing Infection

Although we established that the anti-L2 mAbs are capable of binding HPV L2, binding is not indicative of biological activity or neutralization. Neutralization was tested using pseudovirus (PsV) and 293TT cells, but pre- and post-attachment neutralization by the anti-L2 mAbs was generally incomplete and variable (data not shown). In light of these results, we decided to test the neutralization capacity of Quasivirions (QVs). The 16L1/L2 QVs contained an authentic papillomavirus genome and were used to infect a species matched cell-line, RA2LT [57].

**Figure 4:**
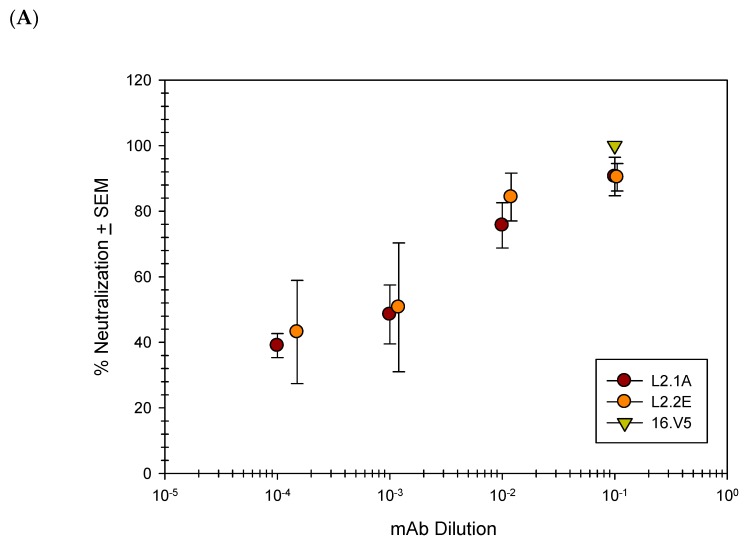

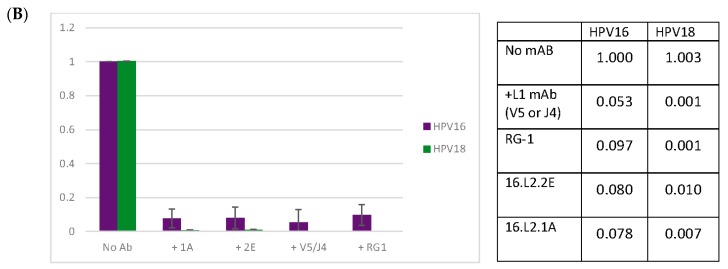
Anti-L2 mAbs neutralize HPV16 virus particles. Neutralizing capacity of anti-L2 mAbs was examined and mean values of three independent assays are shown. Infectivity was determined using a quantitative real-time (qRT)-PCR based assay for the detection of the E1^E4 splice transcript. (**A**) Percent QV16 post-attachment neutralization in RA2LT cells relative to infection without mAb; (**B**) Native HPV16 and HPV18 post-attachment neutralization in HaCat cells relative to infection without mAb. SEM: standard error of the mean.

Previous studies have shown that upon binding to the cellular surface, the capsid undergoes a conformational change and exposes additional L2 residues [28,64,65,66,67,68]. Given the potential for enhanced binding of L2 mAbs following the binding of QV16 to the cellular surface, we proceeded to test the ability of the L2 mAbs to neutralize QV16 in a post-attachment neutralization assay. Nearly complete neutralization was attainable at the highest concentration of mAb with a half maximum neutralization achieved using a 1:1000 dilution (Figure 4A). The anti-L2 mAbs were subsequently tested for their ability to neutralize HPV16 and HPV18 raft-derived native virions (Figure 4B). Neutralization of both HPV types using 1A and 2E was complete and comparable to another previously published anti-L2 mAb, RG-1 [11].

### 3.4. Anti-L1 Capture ELISAs Reveal U4 and Anti-L2 mAb Competition

For the purpose of the competition ELISAs, we chose to use three anti-L1 mAbs, H16.V5 (V5), H16.7E (7E) and H16.U4 (U4), for which the epitopes were previously mapped using loop replacement/hybrid VLPs and/or particle reconstructions [53,54,55,69]. 7E was exclusively mapped to the BC surface exposed variable loop by hybrid binding studies [69]. While V5 preferentially binds the tips of the hexavalent L1 pentamer as seen in reconstructions by Lee et al. (Figures 1 and 7 in [55]), the binding pattern of U4 contrasts sharply. U4 binds the area surrounding pentavalent capsomers at the C-terminus of L1 as demonstrated by reconstructions published by Guan et al., Figure 1 [54]. The C-terminus is localized between capsomers close to the base of the capsid floor [70].

**Figure 5:**
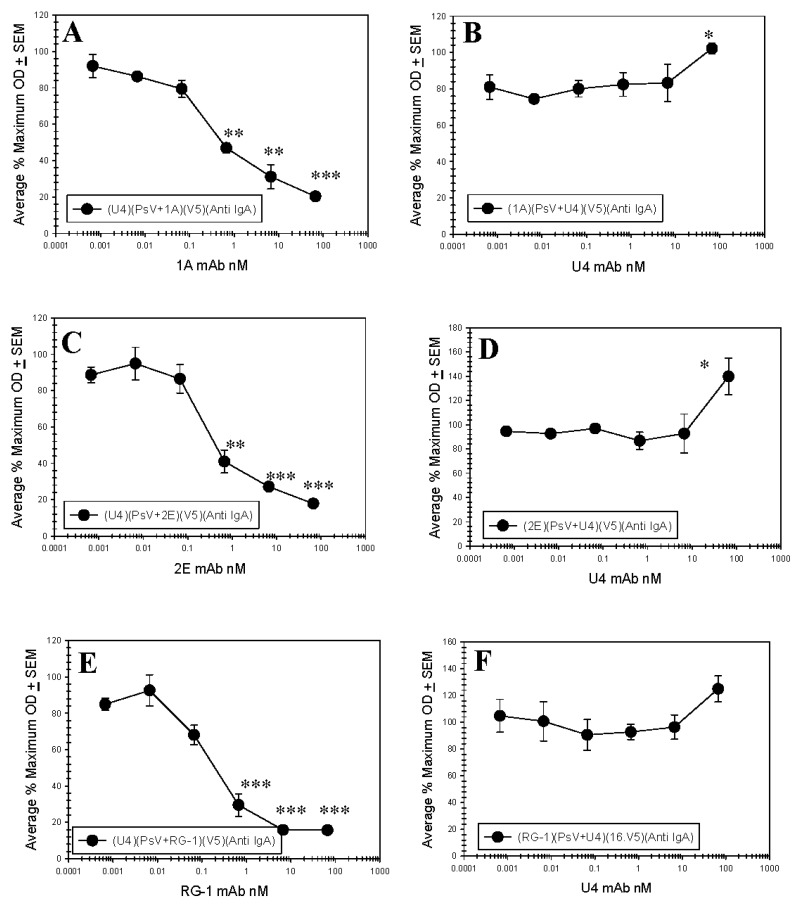
Anti-L1 and Anti-L2 mAb Competition Capture ELISAs. PsV16 pre-incubated with a titration of H16.U4, RG-1, L2.1A or L2.2E were captured in ELISA by another mAb. Captured pseudoviruses (PsVs) were detected with an IgA isotype switch-variant of 16.V5 and an anti-IgA secondary mAb. The % maximum optical density (OD) is reported as an average of three separate assays. A Student’s *t*-test was used to assess the statistical significance of each plotted point relative to the minimum concentration of pre-incubation mAb. *p* ≤ 0.05 *, *p* ≤ 0.01 **, *p* ≤ 0.001 ***. (**A**) PsV pre-incubated with 1A mAb and captured with U4 mAb; (**B**) PsV pre-incubated with U4 mAb and captured with 1A mAb; (**C**) PsV pre-incubated with 2E mAb and captured with U4 mAb; (**D**) PsV pre-incubated with U4 mAb and captured with 2E mAb; (**E**) PsV pre-incubated with RG-1 mAb and captured with U4 mAb; (**F**) PsV pre-incubated with U4 mAb and captured with RG-1 mAb.

To elucidate anti-L2 mAb epitope in the context of assembled virions, a series of competition ELISAs examining the binding of both the novel anti-L2 mAbs and the panel of anti-L1 mAbs were performed. In order to maintain the native conformation of the PsV particles and avoid any potential structural alterations which might occur by direct binding of particles to the plastic of the ELISA plate, a capture method was employed. A schematic diagram of the assay format is shown in Figure S4A,B. Our initial studies examined competition between three anti-L1 mAbs (U4, V4 and 7E) and the novel anti-L2 mAb 2E (Figure S5). U4, V5 or 7E were bound to the wells of a microtiter plate to serve as a capture antibody. PsV16 was pre-incubated with serial dilutions of 2E mAb and subsequently added to the wells. Solution-phase binding of 2E was confirmed by gel filtration chromatography and sequential immunoblot (Figure S6, Appendix A). Since the anti-L2 mAb was pre-incubated with the PsV particles as a titration, we were unable to utilize the anti-L2 mAb as a proxy for captured PsV particles. Therefore, we used an isotype switch-variant of V5 (IgA) and an alkaline phosphate conjugated IgA-specific secondary antibody to assess successful PsV capture. We confirmed by ELISA that neither the V5 IgA detection antibody or the secondary antibody is able to bind the wells of the microtiter plate pre-treated with capture mAb (Figure S7). Capture of the 2E pre-coated PsV by 7E was weakly inhibited by the lowest dilution of 2E mAb (Figure S5). The percent maximum IgA signal was reduced by approximately 44%. Capture by V5 was inhibited intermediately with a difference of 57% and U4 capture was strongly inhibited with the lowest dilution of 2E resulting in a difference of 87% (Figure S5). We further investigated the interference observed between U4 and 2E in addition to 1A and RG-1 using purified mAbs to ensure saturation of viral epitopes (Figure 5). At the highest concentrations, all three anti-L2 mAbs significantly interfered with U4 mAb capture of PsV particles (Figure 5). 

### 3.5. Anti-L2 Capture ELISAs Reveal the Accessibility of the L2 Epitope in the Presence of H16.U4

Given the strong competition observed in the L1 capture ELISAs with the two mAbs U4 and 2E, we next assessed whether the same interference occurred when U4 was first bound to particles. In contrast to the interference observed when capturing PsV-2E mAb with U4 mAb, capture of PsV-U4 mAb using 2E was uninhibited (Figure S5). These assays were repeated with a titration of purified mAb and we also examined the potential for the two other anti-L2 mAbs (1A and RG-1) to interfere. Even at the greatest concentration of U4 mAb, all anti-L2 mAbs successfully captured PsV (Figure 5). 

### 3.6. Direct ELISAs Alter Epitope Availability 

A previous study examining the binding capabilities of mAb RG-1 noted binding to PsV particles when the particles were bound to polystyrene [11] but binding was not achieved in solution [65]. Although in our studies we were able to attain solution-phase binding of RG-1 to PsV, there exists the possibility that a direct interaction with the plastic substrate can alter the structure and influence some of the epitopes presented by the capsid [71,72,73,74,75,76]. In order to explore the possibility that the alternate assay format could have an impact on the results, the very same competition between anti-L1 mAbs and anti-L2 mAbs that was assessed via capture ELISA was also assessed in a direct ELISA. A schematic diagram of the assay is shown in Figure S4C,D. Initial assays were performed using dilutions of 2E mAb supernatants (Figure S5). In the first assay, PsV was bound to the microtiter plates followed by a titration of U4 and a subsequent addition of 2E supernatants at a 1:100 dilution (Figure S5). The presence of bound 2E was determined using an isotype specific secondary antibody. Similar to the results observed in the capture ELISA, the preliminary binding by U4 did not influence the binding of 2E. Contrary to what was observed using the capture ELISA however, preliminary binding by 2E also did not interfere with U4 mAb binding in the direct ELISA. 

**Figure 6:**
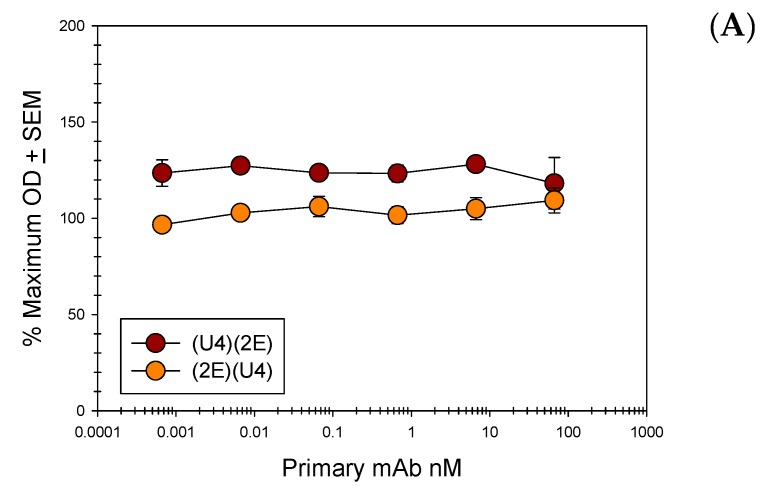

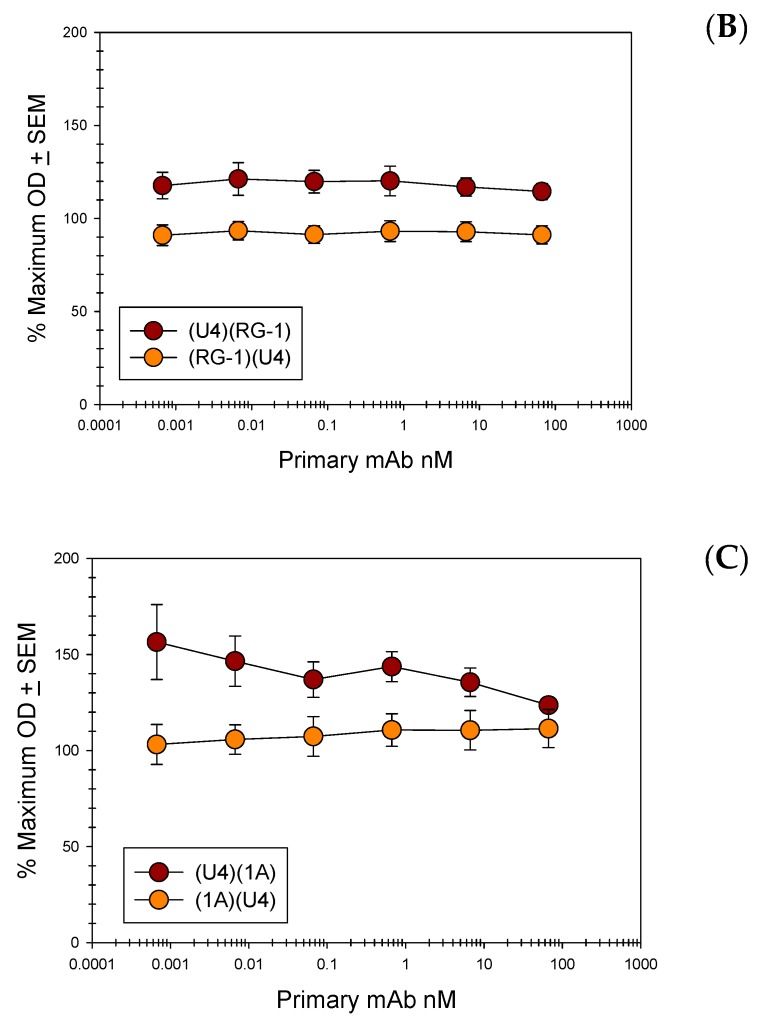
Anti L1 and Anti L2 mAb Direct Binding Competition ELISAs. Pseudovirus 16 particles (500 ng/well) were directly bound to the wells of a microtiter plate. A titration of purified 16.U4 mAb or anti L2 mAb (L2.1A, L2.2E, or RG-1) was added to particles. A constant concentration (66.6 nM) of a reciprocal purified mAb, 16.U4 or an anti L2 mAb, was subsequently added to the virus particles and binding of this antibody was detected with an isotype specific alkaline phosphatase (AP)-conjugated secondary mAb. The % maximum OD is reported as an average of three separate assays. A Student’s *t*-test was used to assess the statistical significance of each plotted point relative to the minimum concentration of titrated primary mAb. None of the *p* values were ≤0.05. (**A**) Direct competition between 16.U4 mAb and RG-1 mAb; (**B**) Direct competition between 16.U4 mAb and L2.2E mAb; (**C**) Direct competition between 16.U4 mAb and L2.1A.

We extended these direct ELISA studies to V5 and 7E mAbs (Figure S5). As expected, 7E mAb binding did not greatly influence the binding of 2E and reduced the signal of the anti-L2 mAb by only 12%. However, binding of V5 to PsV particles interfered with 2E detection by approximately 52%. This level of interference is similar to that observed when interference was assessed by capture ELISA. We then performed a second set of direct ELISAs in which 2E was bound to PsV prior to the anti-L1 mAbs and assessed the ability of the anti-L1 mAbs to bind PsV. Unlike the previous assay, this assay did not detect any interference between anti-L1 and anti-L2 mAbs when anti-L2 mAbs were first bound to the PsV particles (Figure S5). After observing the lack of interference between 2E and U4, we examined whether 1A and RG-1 exhibited the same behavior. Using the same assay, we subsequently tested the ability of purified 2E, 1A and RG-1 to bind their epitopes in the presence of U4 following PsV binding to polystyrene surfaces (Figure 6). The results using purified anti-L2 mAbs mirrored those observed using 2E supernatants. 

### 3.7. L2 Quantification Agrees with Degree of U4 and 2E Competition 

**Figure 7:**
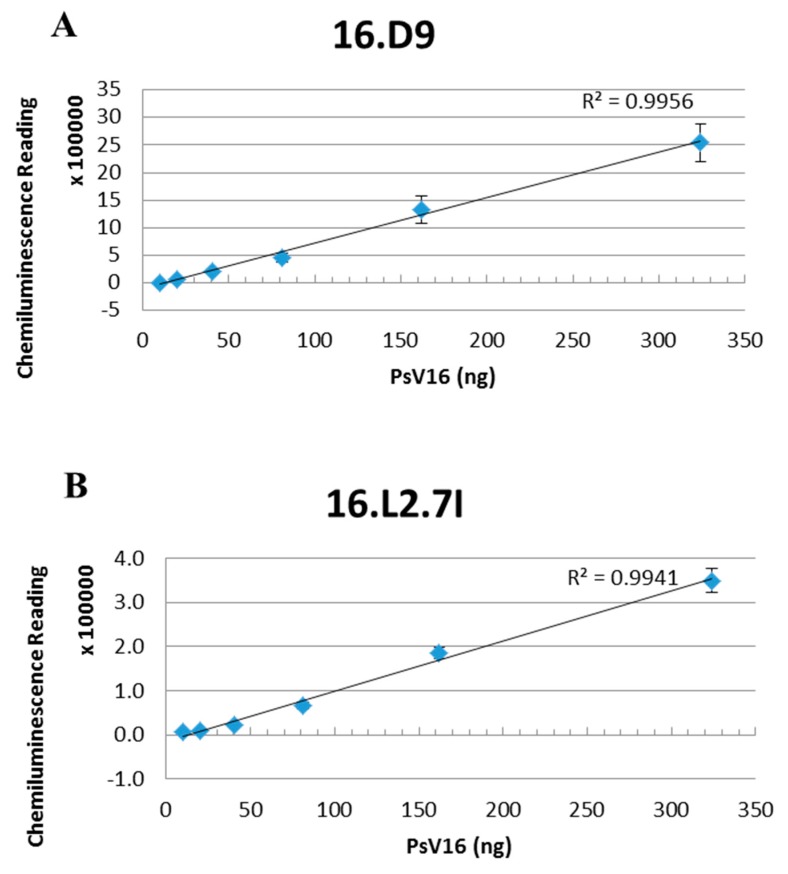
L2 content of PsV preparations. Denatured PsVs were serially diluted and added to the nitrocellulose membrane of the Quantitative Dot Blot (QDB) plate in quadruplicate. L1 and L2 were detected with (**A**) 16.D9 and (**B**) 16.L2.7I mAbs respectively and a secondary horseradish peroxidase HRP conjugated mAb. Plates were developed with enhanced chemiluminescence (ECL) peroxidase substrate and the chemiluminescent signal was read with a plate reader. Three separate assays were performed and representative linear regressions are shown. The ratios at the top three PsV concentrations were averaged to obtain a final ratio of 7:1 or approximately 50 L2 monomers.

Our previous studies have determined that U4 binds the intercapsomeric clefts of pentavalent capsomers [54]. There is a total of 12 pentavalent capsomers and 60 potential binding sites. Therefore, analysis of the interference observed in the capture assay between 2E and U4 estimates 60 copies of L2 incorporated into the capsid. In the capture ELISAs, we never observed complete interference as the highest concentration of anti-L2 mAb resulted in approximately 20% of the maximum capture signal. Failure to completely block capture suggests that there are slightly less anti-L2 epitopes in comparison to U4. To assess the total number of monomers incorporated, we used the Quantitative Dot Blot (QDB) (Figure 7). Serially diluted PsV16 was denatured and probed with anti-L1 mAb H16.D9, which detects a linear epitope, and a third anti-L2 detecting mAb H16.L2.7I (7I) (prepared in-house). 7I mAb was used in the QDB assay because 1A and 2E detected L2 poorly under the denaturing conditions of the QDB assay. Chemiluminescent signals from three independent assays and linear regressions were plotted for each mAb and the L1:L2 ratio was calculated. On average, the QDB assay detected approximately 50 L2 monomers and an L1:L2 ratio of 7:1. When plotted as a normal distribution (Figure S8), 68% of the PsVs contained between 42 and 56 L2 monomers and 95% of the particles contained 36–64 monomers.

## 4. Discussion

In this work, we evaluated two novel anti-L2 mAbs generated against aa17–36 of HPV16 L2 in order to assess L2 location, and neutralization mechanisms. Sequence analysis revealed that the two mAbs generated against the L2 peptide are unique. Peptide binding ELISAs and immunofluorescence assays confirmed that both mAbs bind HPV16 and HPV18 but have individual binding profiles against the other HPV types tested. When we assessed the biological activity of the anti-L2 mAbs, we determined that the mAbs are neutralizing and neutralization of both QV and Native Virus was comparable to RG-1. Finally, we investigated the surface location of L2 aa17–36 through a series of competition assays. To the best of our knowledge, this is the first study to assess the surface location of L2 by examining competitive binding of sets of anti-L1 and anti-L2 mAbs. From these analyses, we describe a surface location of L2 aa17–36 accessible to antibody within intercapsomeric clefts. 

Improved neutralization in a post-attachment neutralization assay was previously documented by Selinka et al. [64] with HPV33 mAbs. In our studies, both 1A and 2E mAbs neutralized QV16 in pre-attachment and post-attachment neutralization assays. We noted that the neutralization efficiency was not as robust in the pre-attachment assay (data not shown) compared to the post-attachment assay but the difference was not statistically significant. Since 1A and 2E were able to neutralize both native HPV and QVs with similar efficiencies as anti-L2 mAb RG-1, we conclude that both HPV particles display the same neutralizing epitopes. 

Both direct binding ELISAs and capture ELISAs were used to examine competition between U4 and 2E. We approached the search for the L2 epitope with caution as we wanted to avoid inducing conformational changes that are not present in the native virion structure in solution phase. An extensive list of previous studies outside of the papillomavirus field have noted differential mAb binding in various ELISA formats and have reported that direct coating of the microtiter plates with the antigen was found to be the least reliable method for assessing antibody binding [76,77]. Direct binding of antigen to the polystyrene surfaces induces conformational changes in the protein and can also alter the antigenicity [72,73,76,77,78,79,80,81,82]. An example of induced conformational changes to HPV was observed with RG-1. RG-1 binds PsV in standard ELISA and protects against PsV infection in vivo [11]; however, RG-1 does not detect PsV in solution [65]. Day et al. [65] speculated that binding of the capsid to the plastic alters the conformation of the capsid and reveals the otherwise hidden RG-1 epitope. In contrast, the binding of other mAbs such as V5 are not influenced by any conformational changes that may arise due to the direct binding of antigen to plastic and continues to recognize the viral antigen. We must remember that conformational changes are also the mechanism postulated for viral entry and the very same conformational changes that occur on the cell surface may also be initiated by direct binding of capsids to the microtiter plates. 

In an attempt to detect epitopes in the most conformationally native state as possible, we assessed competition between mAbs using a capture ELISA. PsV particles pre-coated with anti-L2 mAb were unable to be efficiently captured by U4. However, PsVs pre-coated with U4 were able to be efficiently captured by 2E, revealing that the two antibodies do not compete when U4 is first bound to the PsV. As we suspected, direct binding ELISAs did not recapitulate the results obtained by capture ELISA such that there was not any competition observed between U4 and 2E regardless of the order of addition of the two mAbs. 

It is probable that the use of a whole mAb as opposed to a Fab or even (Fab)_2_ results in increased steric interference and clashing of the Fc regions between mAbs. It would have been beneficial to confirm our results using Fab fragments, but the production of Fabs from 1A to 2E has not been successful for unknown reason. The production of other anti-L2 Fabs has also proven to be technically challenging. Although the contribution to steric interference by the Fc fragment has not been determined, based upon the exaggerated decrease in particle capture with U4, our primary hypothesis is that the epitopes of U4 and 2E share a similar location on the capsid. The known U4 epitope at the C-terminal arm of the fivefold vertex [54] leads us to propose that portions of the L2 minor capsid protein (aa17–36) are located between capsomers surrounding the pentavalent capsomer. These results do not eliminate the possibility that 2E also binds in the canyons of hexavalent capsomers. A failure of U4 and 2E to compete when U4 was bound to PsV first could suggest that U4 has an epitope which is more deeply buried in the canyon of the capsid. We hypothesize that in contrast, the 2E epitope is located closer to the top of the capsid canyon and is therefore successful at blocking U4 from binding its epitope at the base of the canyon. Since we know that U4 binds exclusively at the pentavalent canyons [54], an alternative scenario is that the 2E epitope is located at both canyons of the pentavalent and hexavalent capsomers. In such a scenario, 2E would capture and bind virus particles in areas where U4 is not binding and thus completely avoid steric interference in those areas. This observation could explain why PsVs are able to be captured by 2E in the presence of U4. Further, if 2E is able to bind non-discriminately between pentavalent and hexavalent capsomers alike, this could explain why U4 capture of particles in the presence of 2E is hindered. 

Although the capture ELISA method was intended to analyze the capsid structure in its conformationally correct state, we cannot dismiss the possibility that the capture mAb induces a structural change in the viral capsid. Such putative structural changes could be local or global but also could ultimately alter the availability of epitopes and modify the ability of other mAbs to bind viral capsids. Specifically, a scenario exists whereby U4 binding exposes the 2E epitope. While we cannot at this point unravel the finer intricacies of viral capsid activation, we are encouraged that these mAbs as well as new, yet to be characterized molecular probes may be able to be used to explore these questions in future studies. 

Previous studies have hinted at similar epitopes for U4, heparin, and L2, but to the best of our knowledge proximal U4 and L2 epitopes have never been directly proposed. Day et al. [83] investigated the capsid binding pattern in the presence of either V5 or U4. Particles pre-incubated with V5 were able to bind to the cell surface but not the extracellular matrix (ECM). U4 pre-incubated particles were sequestered to the ECM and were prevented from binding the cellular surface indicating that U4 interferes with engagement of the virus particles and cellular receptors [83]. Subsequent studies demonstrated that both RG-1 and heparin also prevent binding to the cellular surface [65]. Similar behavior by U4 and RG-1 suggests that the two epitopes are present in the same general location of the capsid. 

Further evidence in support of our hypothesized L2 aa17–36 localization comes from studies using xenograft derived HPV16. Despite demonstrating neutralization of PsV16 with U4, White et al. [84] were unable to neutralize HPV16 from xenografts with U4. Combined with the evidence from Conway et al. [85] that L2 becomes more exposed throughout the virus maturation process, the L2 epitope likely occludes the U4 epitope in fully matured virions. The observation that U4 shows high levels of binding to VLPs but decreased binding to PsV [86] is additional biological evidence in support of this hypothesis. Our recent structural studies utilizing cryo-EM reconstruction techniques localized putative L2 densities [87] that also align with our current biochemical and immunological studies. 

The failure to recapitulate, in the direct ELISA, the competition observed in the capture ELISA between anti-L2 mAbs and U4 further substantiates the observations made by Day et al. [65]. The findings suggest that some of the capsid epitopes are altered upon binding to plastic perhaps even mimicking the conformational changes that occur upon binding to the cell surface, while others remain unchanged or still recognizable by select mAbs. A lack of interference between U4 and anti-L2 mAbs suggests that upon binding, L2 is more accessible possibly due to a more relaxed capsid structure and therefore enables increased binding by anti-L2 mAbs. Although U4 and anti-L2 mAbs did not compete with each other in the direct ELISA, there was interference observed between V5 and 2E (Figure S5). When V5 was bound to the particles first, followed by 2E we observed a 52% decrease in the anti-L2 mAb signal at the lowest dilution. This magnitude of interference is similar to that observed when particles were captured by V5 and therefore it is probable that alterations which occur following binding to plastic do not significantly impact the V5 epitope. However, we questioned why the anti-L2 signal plateaus rather than continues on a downward trend with increasing concentrations of V5. Previous incubations of QV with V5 and cryo-EM reconstructions have identified the V5 epitope at the vertex of hexavalent capsomers [55]. This epitope location is spatially different from U4 and the competition observed between V5 and 2E is not likely to be a consequence of competition for the same epitope. Rather, we suggest that there is a steric clashing of the Fc portions from each mAb. 

It is clear from these studies that the L2 epitope is present in all forms of in vitro produced particles. A semi-quantitative analysis of the L1:L2 ratio of the PsVs used in our assays revealed an average 7:1 ratio among different dilutions signifying an average of 50 incorporated L2 monomers, but the values ranged from 40 to 60 L2 monomers (Figure 7). To date, no assays address whether or not there is a threshold of L2 incorporation. Several questions related to this concept are as follows: (i) is there a minimum threshold number of L2 molecules that must be incorporated into the virus particle to allow for the successful encapsidation of viral or plasmid DNA? (ii) is there a minimum number of L2 molecules per capsid needed for successful viral binding and entry? If there are subthreshold numbers of L2 monomers present in some particles when conducting the neutralization assay, then the number of epitopes available to bind neutralizing antibody will be limited and neutralization escape in the presence of L2 N-mAbs could occur. Variable incorporation of L2, in addition to the requirement for capsid conformational changes, may have contributed to incomplete neutralization in our pre-attachment neutralization assays. In the ELISA, even if there is a small number of L2 molecules present in the particle the signal is amplified due to the use of an anti-L1 for detection of captured particles. Pieces of particles, capsomers, and even empty particles can also be captured by ELISA. In contrast, the read-out in a neutralization assay is the successful delivery of encapsidated DNA such that neither pieces of particles nor empty capsids can contribute to an infectious readout. 

We show in this study that there is great potential for antibodies targeting L1 and L2 to compete with one another. This observation will become increasingly important upon the advent of an L2 vaccine. Competition will specifically need to be considered upon utilizing diagnostic tests such as the competitive response luminex immunoassay (cLIA) for determining patient responsiveness. Although these studies do not definitively address the L1:L2 ratio of patient derived virions, the interference observed between U4 and anti-L2 mAbs indirectly assesses the representation and location of L2 aa17–36. If there is a single L2 epitope for every U4 epitope, then it would estimate 60 L2 proteins present in an average PsV particle which is fairly consistent with our quantitation studies (Figure 7). In future work, we aim to examine the functional effects of the anti-L2 mAbs on furin cleavage as Bronnimann et al. [88] showed that V5 blocks cleavage of L2 while U4 did not have this effect. We acknowledge that structural data would be complementary to our biochemical assays presented herein. While Fabs are favorable in such reconstructive studies due to their decreased flexibility compared to mAbs, as previously mentioned we are not at this time able to successfully process usable Fabs. We have therefore conducted preliminary cryo-EM studies using whole 2E mAbs, but we have not yet been able to resolve the density of the mAb in reconstructions. In order to definitively target and image the minor capsid protein, our future plans will include improved cryo-EM techniques paired with alternative anti-L2 Fabs or scFVs. Given the difficulty previously experienced in processing anti-L2 mAbs into Fabs, we are currently working with a larger panel of anti-L2 mAbs in addition to 2E and 1A described in this study. 

## Figures and Tables

**Table 1:** Summaryof the results from the peptide ELISA and immunofluorescence (IF) assay.

		Peptide ELISA			IF	
	Peptide Sequence aa17—36	RG-1	H16.L2.1A	H16.L2.2E	H16.L2.1A	H16.L2.2E
HPV16 L2	QLYKTCKQAGTCPPDIIPKV	++++	++++	++++	+	+
HPV18 L2	DLYKTCKQSGTCPPDVVPKV	++++	++	++	+	+
HPV31 L2	QLYQTCKAAGTCPSDVIPKI	+	−	−	NT	NT
HPV35 L2	QLYRTCKAAGTCPPDVIPKV	+++	+	−	±	−
HPV39 L2	DLYRTCKQSGTCPPDVVDKV	+	−	+	−	+
HPV45 L2	DLYRTCKQSGTCPPDVINKV	+	−	+	−	+
HPV58 L2	QLYQTCKASGTCPPDVIPKV	+	−	−	NT	NT
HPV59 L2	DLYKTCKQAGTCPSDVINKV	++++	+	−	+	−

The HPV type and its corresponding L2 sequence aa17—36 is presented on the left. Peptide binding is rated by a series of (+) symbols, where (++++) indicates strong positivity, (+) indicates weak positivity, and (−) indicate a lack of binding. Positivity by IF is rated as either positive (+), weak (±), or negative (−). NT: Not Tested.

## Supplementary Materials

The following are available online at www.mdpi.com/1999-4915/9/11/336/s1.

## Appendix A

### Gel Filtration Chromatography and Immunoblot

PsV16 was incubated in solution for 1 h at room temperature with sufficient biotinylated 2E mAb to bind 30 hypothetical epitopes per viral capsid or biotinylated 2E was incubated alone in an equal volume of PBS. PsV/mAb or mAb were added to a 2 mL column of 2% agarose beads (Agarose Bead Technologies, Doral, FL, USA) and eluted with DPBS/0.5 M NaCl [38,89]. Each fraction contained 250 L eluate and 15 fractions in total were collected. Fractions 1–15 (5 µL) were separated by SDS-PAGE on a 10% Criterion TM TGXTM Precast gel (Bio-Rad), transferred to a PVDF membrane, and probed with antibodies. The L1 capsid protein was detected by H16.D9 mAb supernatant (1:100) and a goat anti-mouse IgG1 HRP conjugated secondary (1:5000) (Invitrogen A10551). The L2 capsid protein was detected using anti-L2 rabbit polyclonal serum (1:50) [11,90] and swine anti-rabbit HRP (1:15,000) (Dako-Agilent P0399, Santa Clara, CA, USA). Biotinylated 2E mAb was probed using streptavidin HRP (1:2500) (Invitrogen 43-4323, Carlsbad, CA, USA). Secondary antibodies were detected using Chemiluminescent FemtoMaxTM Super Sensitive HRP Substrate (Rockland, Limerick, PA, USA) and blot images were obtained with a FluorChem R machine (ProteinSimple, San Jose, CA, USA).
