# Supplementary material for: Antibody Competition Reveals Surface Location of HPV L2 Minor Capsid Protein Residues 17–36
Source: Viruses. 2017 Nov 10;9(11):336. doi: 10.3390/v9110336 (PMC5707543; doi:10.3390/v9110336)
Supplement: Supplementary file 1 [file viruses-09-00336-s001.pdf]

Supplemental Figures

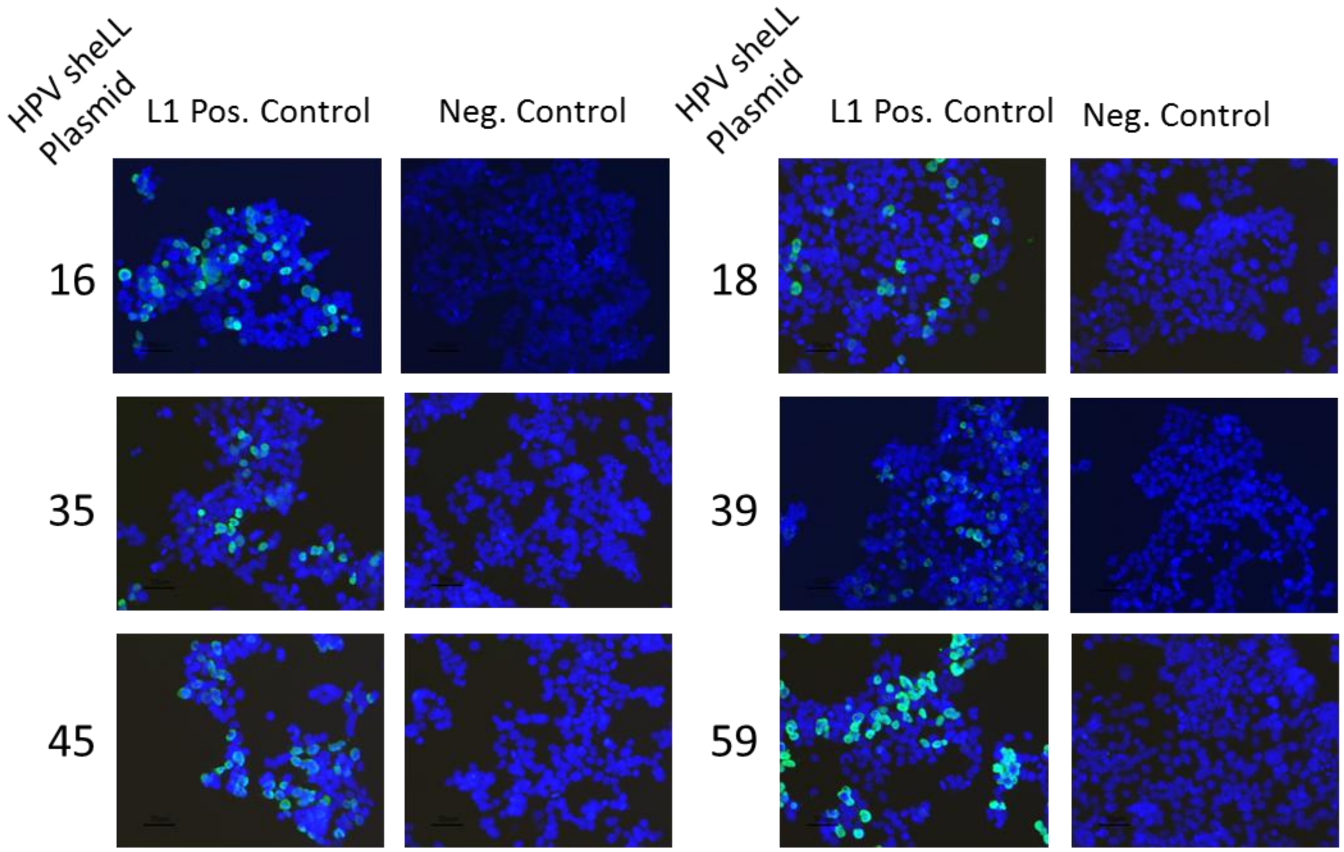

Figure S1. 293TT cells were seeded at a density of $2x10^5$ on poly-L-lysine treated glass coverslips and cultured overnight. Cells were transfected with HPV sheLL plasmids using Lipofectamine 2000 (LifeTechnologies) and cells were left to culture for an additional 48 hours. Cells were fixed with acetone:methanol, blocked with 2% bovine serum albumin (BSA) in phosphate buffered saline supplemented with Tween-20 (PBS/T), incubated with a 1:100 dilution of primary monoclonal antibody (mAb), and stained with a 1:1000 dilution of goat anti-mouse IgG (H + L) 488 conjugated secondary mAb and Hoescht (ThermoFisher Scientific). The following mAbs were used as positive controls: HPV16 sheLL (H16.V5), HPV18 sheLL (H18.J4), HPV35 sheLL (35.Q8), HPV39 sheLL (39.B6), HPV45 sheLL (45.N5), HPV59 sheLL (59.G1). Negative Controls were transfected with sheLL plasmids and incubated with secondary mAb and Hoescht.

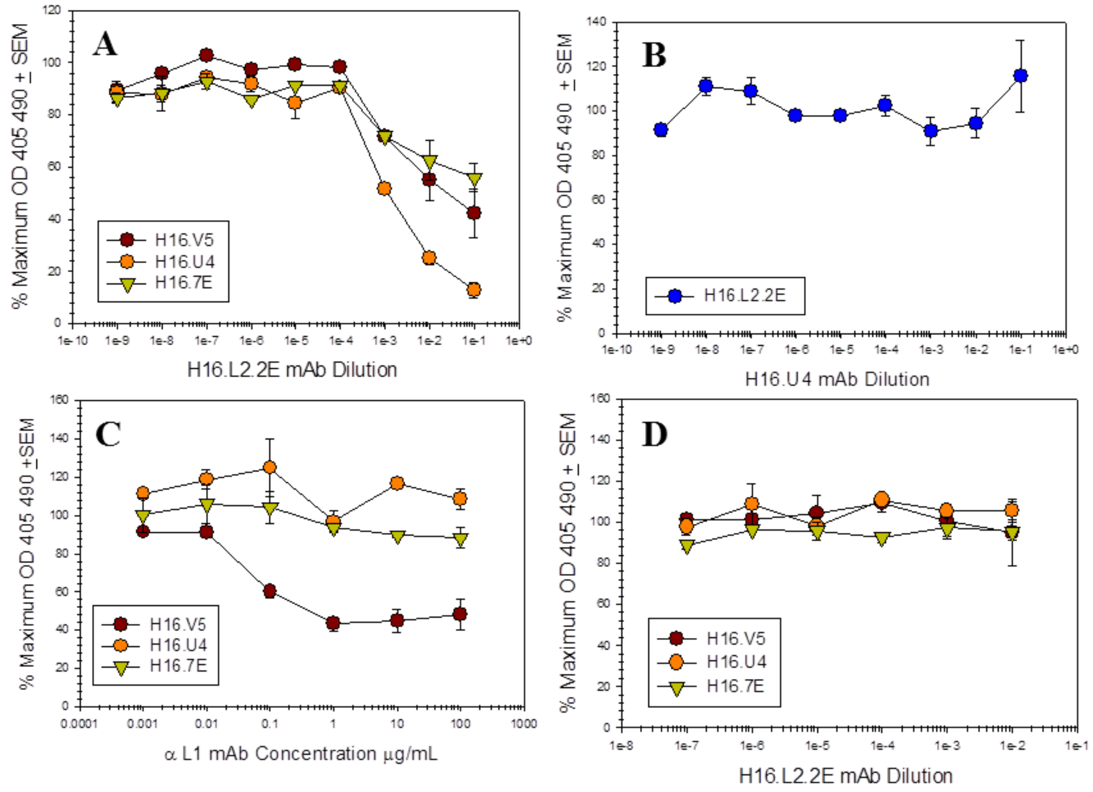

Figure S2. **(A)** H16.L2.2E interferes with Pseudovirus (PsV) capture by anti-L1 mAbs. Anti-L1 mAbs were bound to the wells of a microtiter plate and used to capture PsV pre-incubated with serial dilutions of H16.L2.2E. Capture efficiency was determined as a percent of the maximum H16.V5 IgA signal and displayed as an average of two separate assays. **(B)** H16.U4 does not interfere with PsV capture by anti-L2 mAb. PsV pre-incubated with serial dilutions of H16.U4 were captured by H16.L2.2E. Detection of bound particles was assessed by H16.V5 mAb isotype IgA. The IgA signal was averaged between two separate assays and plotted as a percentage of the maximum signal. **(C)** PsV particles were bound directly to the plastic wells of a microtiter plate. A serial dilution of anti-L1 mAbs were added first followed by H16.L2.2E. Successful binding of the anti-L2 mAb was determined using an isotype specific secondary antibody. **(D)** PsV particles were bound directly to the plastic wells of a microtiter plate.

A serial dilution of H16.L2.2E was added first followed by anti-L1 mAbs. Successful binding of the anti-L1 mAbs was determined using isotype specific secondary antibodies.

Figure S3

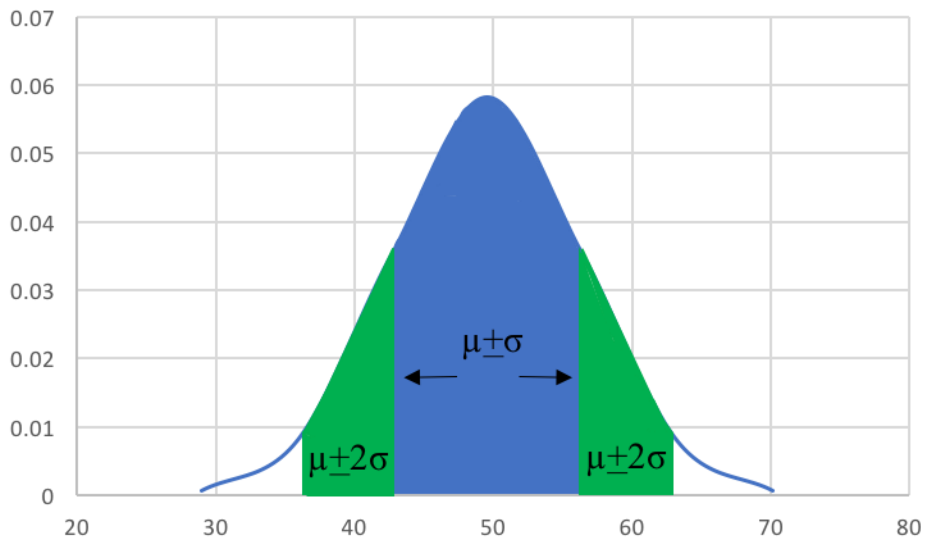

Figure S3. The averaged data from all three Quantitative Dot Blot (QDB) assays was used to compute a normal distribution of L2 monomers per PsV capsid. The area under the curve shaded in blue represents one standard deviation from the mean and the number of L2 monomers contained in 68% of PsV particles. The area under the curve shaded in green represents two standard deviations from the mean and the number of L2 monomers contained in 95% of PsV particles.
